# Comparison of Two Malnutrition Assessment Scales in Predicting Postoperative Complications in Elderly Patients Undergoing Noncardiac Surgery

**DOI:** 10.3389/fpubh.2021.694368

**Published:** 2021-06-21

**Authors:** Fang Zhang, Shu-Ting He, Yan Zhang, Dong-Liang Mu, Dong-Xin Wang

**Affiliations:** Department of Anesthesiology, Peking University First Hospital, Beijing, China

**Keywords:** malnutrition, Nutritional Risk Screening 2002 (NRS2002), perioperative nutrition screen (PONS), postoperative complications, elderly patient

## Abstract

**Background:** The present study was designed to investigate the relationship between two malnutrition assessment scales, perioperative nutrition screen (PONS) and Nutritional Risk Screening 2002 (NRS2002), with postoperative complications in elderly patients after noncardiac surgery.

**Methods:** This was a secondary analysis of a prospective cohort study. Elderly patients (65–90 years) undergoing noncardiac surgery were enrolled in Peking University First Hospital. Malnutrition was screened by PONS and NRS2002 at the day before surgery. Multivariable analysis was employed to analyze the relationship between PONS and NRS2002 and postoperative 30-day complications. Receiver operating characteristic (ROC) curve was generated to evaluate the predictive value of PONS and NRS2002 in predicting postoperative complications.

**Results:** A total of 915 patients with mean age of 71.6 ± 5.2 years were consecutively enrolled from September 21, 2017, to April 10, 2019. The incidence of malnutrition was 27.3% (250/915) by PONS ≥ 1 and 53.6% (490/915) by NRS2002 ≥ 3. The overall incidence of complications within postoperative 30 days was 45.8% (419/915). After confounders were adjusted, malnutrition by PONS ≥ 1 (OR 2.308, 95% CI 1.676–3.178, *P* < 0.001), but not NRS2002 ≥ 3 (OR 1.313, 95% CI 0.973–1.771, *P* = 0.075), was related with an increased risk of postoperative complications. ROC curve analysis showed that the performances of PONS [area under the ROC curve (AUC) 0.595, 95% CI 0.558–0.633] showed very weak improvement in predicting postoperative complications than NRS2002 score (AUC 0.577, 95% CI 0.540–0.614).

**Conclusion:** The present study found that malnutrition diagnosed by PONS was related with an increased risk of postoperative complications. The performances of PONS and NRS2002 were poor in predicting overall postoperative complications.

**Clinical Trial Registration:** www.chictr.org.cn, identifier: ChiCTR-OOC-17012734.

## Introduction

Malnutrition has been considered as a great challenge to patient's safety in perioperative settings (1, 2). It is estimated that 24–51% of surgical patients are at risk of malnutrition (3, 4). The incidence of malnutrition reaches up to 60–86% in the elderly (4, 5). Malnutrition is associated with increased risk of morbidity (i.e., gastrointestinal fistula, wound dehiscence, and infection) and mortality (1–5). Nutrition support in malnourished patients can reduce the risk of postoperative complications such as surgical site infections and gastrointestinal complications (6–8). Selection of proper assessment tools is the key step for early diagnosis and treatment of malnutrition (9).

Perioperative malnutrition can be mainly attributed to inadequate food intake, decreased physical activity, and catabolic metabolic derangements (1, 2). But manifestations of malnutrition vary greatly among surgical patients such as body weight loss, skeleton muscle mass loss, inflammatory response, low serum albumin, and micronutrient insufficiency (i.e., vitamin D) (1, 2). Several terms, “sarcopenia,” “cachexia,” and “myostetosis,” have been advocated to describe the different characteristics and nutritional syndromes of malnourished patients (10). Although the underlying definition of malnutrition phenotypes is complex and challenging, several assessment instruments have been proposed to facilitate clinical diagnosis of malnutrition based on the following core criteria: body mass index (BMI), body weight loss, serum albumin, and oral food intake (1, 2).

There are dozens of tools to assess nutritional status for metabolic care or clinical nutrition purposes (11). Utility of these instruments should be based on patient population. For example, patient-generated subjective global assessment was generated in cancer patients, and geriatric nutritional risk index was used for patients with heart failure (12, 13). In surgical patients, perioperative nutrition screen (PONS) and Nutritional Risk Screening 2002 (NRS2002) are recommended to screen malnutrition (1, 10). There are insufficient data to elucidate their associations with clinical outcomes in elderly Chinese patients.

PONS is developed and proposed by the American Society for Enhanced Recovery for preoperative screening of malnutrition in 2018 (1). PONS is a modified version of the malnutrition universal screening tool and determines the presence of nutrition risk based on BMI, recent body weight loss, decrement of dietary intake, and preoperative albumin concentration (1, 14). NRS2002 is another assessment tool that has been widely validated in perioperative settings (15). Compared with PONS, NRS2002 includes the severity of disease as supplemental parameter (1, 15). In patients undergoing gastrointestinal surgery and hip fracture surgery, malnutrition (diagnosed by NRS2002 ≥ 3) is highly related with increased risk of postoperative complications, prolonged in-hospital stay, and mortality (16, 17). However, up to now, there is lack of evidence to illustrate the relationship between PONS and postoperative complications in the elderly surgical patients and which instrument (PONS or NRS2002) has better performance in predicting the risk of postoperative complications.

The present study was designed to investigate the association between two malnutrition assessment scales, PONS and NRS2002, with postoperative complications in elderly patients after noncardiac surgery.

## Methods

The present study was a secondary analysis of a prospective observational study. The ethical approval for this study was provided by the Clinical Research Ethics Committee of Peking University First Hospital (Chairperson Prof Guo Xiaohui) on August 4, 2017 [2017 (1419), Beijing, China], and registered with Chinese Clinical Trial Registry on September 19, 2017 (ChiCTR-OOC-17012734). Written informed consent was obtained from all participants or their legal representatives.

### Participants

Elderly patients (aged 65–90 years) were included if they were scheduled to undergo noncardiac surgery with an expected duration ≥2 h under general anesthesia. Patients who met any of the following criteria were excluded: (1) refused to participate in the study; (2) previous history of schizophrenia, epilepsy, Parkinson's disease, or myasthenia gravis; (3) unable to communicate due to severe dementia, being comatosed, or language barrier; (4) traumatic brain injury or neurosurgery; or (5) an American Society of Anesthesiologists (ASA) classification of IV or above.

### Anesthesia and Perioperative Management

All patients received standard monitoring on arrival in the operating room including electrocardiogram, noninvasive blood pressure, pulse oxygen saturation, and urine output. During general anesthesia, end-tidal carbon dioxide, expired concentration of inhalational anesthetics, and bispectral index (BIS) were also monitored. Invasive arterial pressure and central venous pressure were used when necessary.

Induction of general anesthesia was completed by propofol and/or etomidate, opioids (sufentanil and/or remifentanil), and muscle relaxants (rocuronium or cisatracurium). Anesthesia was maintained with propofol infusion and/or sevoflurane inhalation. Nitrous oxide could be used as supplementary when necessary. Muscle relaxants were administered when considered necessary. The target depth of general anesthesia was to maintain BIS between 40 and 60.

Muscle relaxants were stopped for at least 30 min before the end of surgery; propofol infusion and sevoflurane inhalation were decreased or stopped according to BIS monitoring; sufentanil was administered when considered necessary. At the end of surgery, residual neuromuscular blockade was reversed with 0.05 mg/kg of neostigmine and 0.02 mg/kg of atropine. Patients were extubated when they met the following criteria: (1) easy to wake up; (2) sufficient reflexes that protect the airway; (3) adequate gas exchange (respiration rate 10–30 breaths per minute and tidal volume > 6 ml/kg); and (4) acceptable hemodynamic status (systolic blood pressure ≥ 90 mmHg and heart rate ≤ 100 beats per minute).

As a routine practice, patients were transferred to the post-anesthesia care unit (PACU) after extubation. Patients were monitored in PACU for at least 30 min and then transferred to the general ward when the Aldrete score was higher than 9.

### Nutrition Assessment by Perioperative Nutrition Screen

The criteria of PONS include the level of preoperative albumin and the following three questions: (1) Does the patient have a low BMI <18.5 kg/m^2^ (<20 kg/m^2^ for patients > 65 years old)? (2) Has the patient experienced weight loss > 10% in the past 6 months? and (3) Has the patient had a reduced oral intake by >50% in the past week? (1, 18). Patients who had any positive response to the three questions and/or serum albumin <30 g/L were considered as at high risk of malnutrition.

### Nutrition Assessment by Nutritional Risk Screening 2002

NRS2002 contains two components: undernutrition and disease severity, giving a total score of 0–6 (15). Undernutrition was estimated by using BMI, percent of recent weight loss, and change in food intake. Each item of impaired nutritional status was classified into absent, mild, moderate, and severe with relevant score of 0–3, respectively. Disease severity is a reflection of stress metabolism, which is divided into normal to severe status with score of 0–3. For example, patients with chronic diseases (i.e., diabetics) are considered as mild grade with a score of three; patients undergoing abdominal surgery are considered as moderate grade with a score of two; and patients with head injury are considered as severe grade with a score of three. Patients are classified as being at nutritional risk when the total score was three or above.

### Postoperative Complications

Postoperative complications are defined as new-onset events that have adverse effect on patient's clinical outcome and need medical treatment (i.e., Clavien-Dindo classification grade II or above) (19). Major complications are listed in Table 1 and include the following items: central nervous system (delirium and stroke), cardiovascular system (myocardial infarction, new-onset arrhythmia, cardiac failure, and pulmonary embolism), respiratory system (pneumonia and respiratory failure), acute kidney injury, surgery-related complications (intestinal obstruction, anastomotic fistula, and bleeding), infection (sepsis, abdominal abscess, and incision infection), and death.

**Table 1:** Frequency and definitions of major postoperative complications.

**Variables**	**Number of all patients (*N* = 915)**
**Number of patients with complications**, ***n*** **(%)**	419 (45.8%)
**Individual complication**, ***n*** **(%)**	
**Central nervous systems**	
Delirium^**a**^	386 (42.1%)
Stroke^**b**^	6 (0.7%)
**Cardiovascular system**	
Myocardial infarction^**c**^	20 (2.2%)
New-onset arrhythmia^**d**^	25 (2.7)
Cardiac failure^**e**^	20 (2.2%)
Pulmonary embolism^**f**^	1 (0.1%)
**Respiratory system**	
Pneumonia^**g**^	13 (1.4%)
Respiratory failure^**h**^	16 (1.7%)
Acute kidney injury^**i**^	26 (2.8%)
**Surgery-related complications**	
Intestinal obstruction^**j**^	3 (0.3%)
Anastomotic fistula^**j**^	11 (1.2%)
Unexpected surgical bleeding^**j**^	3 (0.3%)
Gastrointestinal bleeding^**j**^	7 (0.8%)
**Infection**	
Sepsis^**k**^	9 (1.0%)
Abdominal abscess^**j**^	7 (0.8%)
Incision infection^**l**^	16 (1.7%)
**Death**	13 (1.4%)

a *Delirium was established when the patient suffered any episode of delirium after surgery. According to onset time, it is divided into emergence delirium [from anesthesia emergency to discharge of post-anesthesia care unit (PACU)] and postoperative delirium (from discharge of PACU to postoperative 5 days). Emergence delirium happened in 282 (30.8%) patients and postoperative delirium in 47 (5.1%). A total of 57 (6.2%) patients suffered both emergence and postoperative delirium*.

b *Confirmed by imaging examination and diagnosed by a neurologist*.

c *Cardiac troponin I concentration met the criteria for clinical diagnosis, or ECG showed new emerging Q-waves lasting longer than 0.003 s, or ST-T changes lasting longer than 4 days*.

d *Confirmed by ECG and needed medical treatment, i.e., medicine or electrical cardioversion*.

e *Requiring the use of inotropic agents and/or vasopressors ≥ 24 h to maintain hemostasis*.

f *Diagnosed by clinical presentation and/or imaging examination, i.e., CT or angiography*.

g *New-onset infiltration on chest radiographs, body temperature ≥ 38°C, and white blood cell (WBC) elevated*.

h *Respiratory failure was defined as mechanical ventilation ≥ 24 h*.

i *Serum creatinine increased 1.5–1.9 times baseline or >0.3 mg/dl*.

j *Confirmed by clinical symptoms, imaging examinations, or surgery*.

k *Systemic inflammatory response involved two or more than two systems, existed in at least one organ system dysfunction, or required the use of vasopressors to maintain hemostasis*.

l *Diagnosed by clinical symptoms or positive bacteria culture*.

### Data Collection and Postoperative Follow-Up

Data collection was performed after obtaining written informed consent. Baseline data included demographics, surgical diagnosis, comorbidities, preoperative medication, smoking, Charlson Comorbidity Index, laboratory test results, and ASA classification.

Emergence delirium was defined as delirium that occurred during PACU stay and was assessed using the Confusion Assessment Method for the Intensive Care Unit (CAM-ICU). Postoperative delirium was defined as delirium that occurred in the general ward during postoperative days 1 to 5 and was assessed using the CAM twice daily (8:00–10:00 a.m., 6:00–8:00 p.m.) (19). Pain severity was assessed with the numerical rating scale (NRS; an 11-score scale, with 0 representing no pain and 10 representing severe pain) at the same time interval as that of delirium.

Complications within postoperative 30 days were recorded. From the sixth day after surgery, patients were followed up weekly until postoperative day 30 for the occurrence of postoperative complications. For those who were discharged from the hospital, follow-ups were performed by telephone interview.

### Statistical Analysis

Normality of continuous data was tested by the Kolmogorov–Smirnov method in prior. Data with normal distribution were presented as mean ± standard deviations (SDs), and differences between groups were compared by independents sample *t*-test. Data without normal distribution were presented as median [interquartile range (IQR)], and differences between groups were compared by the Mann–Whitney U test. Categorical data were presented by number (percentage), and differences between groups were compared by chi-square test.

The relationship between NRS2002 and PONS and postoperative complications was firstly analyzed by univariate analysis, followed by multivariable logistic regression analysis adjusted for confounding factors including the baseline characteristics and perioperative variables that showed an imbalance between patients with and without postoperative complications (i.e., *P*-value < 0.05).

Receiver operating characteristic (ROC) curves were used to evaluate the predictive ability of PONS and NRS2002 against postoperative complications.

Two-sided *P* < 0.05 was considered as statistically significant. Statistical analysis was performed using SPSS 26 Inc (Chicago, IL, USA).

## Results

### Patients

**Figure 1:**
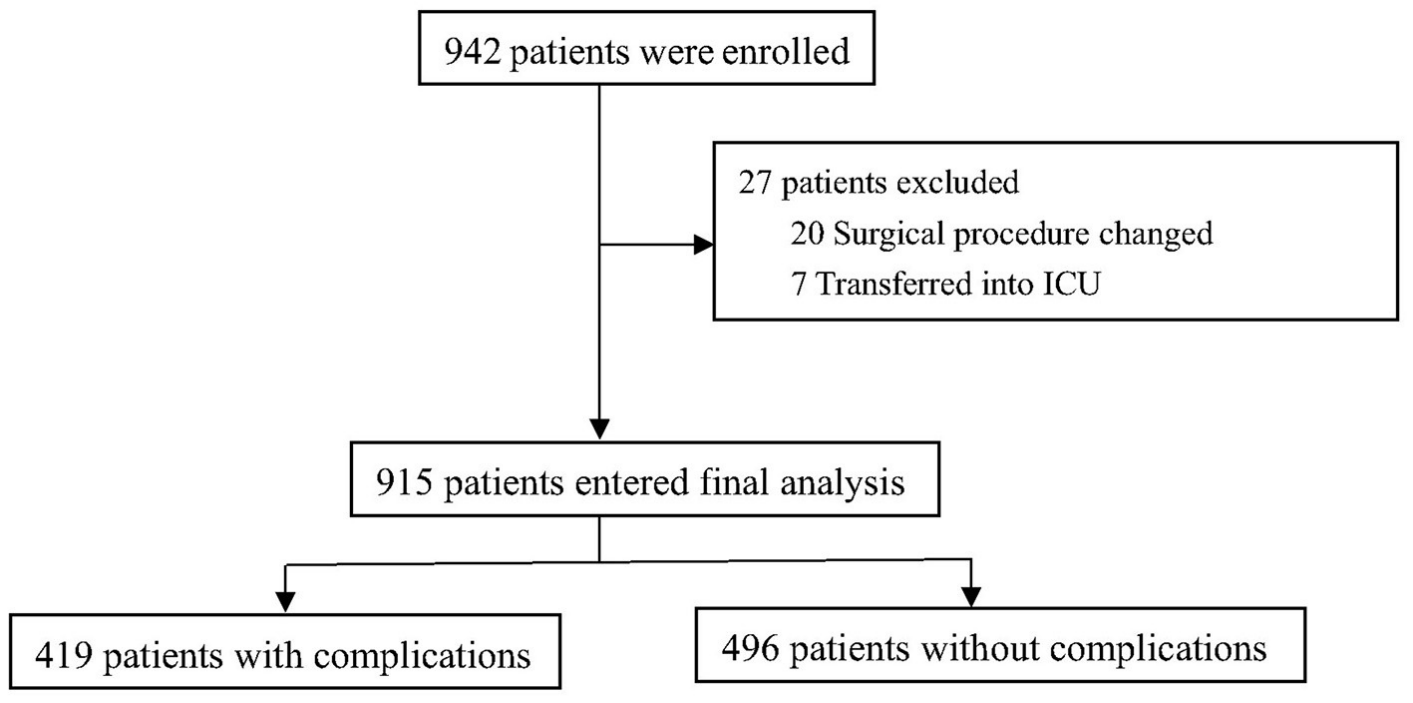
Flowchart of the study.

From September 21, 2017, to April 10, 2019, 942 patients were enrolled and 915 patients were included with mean age of 71.6 ± 5.2 years (Figure 1). The 30-day all-cause mortality was about 1.4% (13/915).

Overall incidence of postoperative complications was about 45.8% (419/ 915), and the incidence of individual complication is listed in Table 1.

In comparison with patients without complications, the mean age was higher in patients with complications (72.7 ± 5.7 vs. 70.7 ± 4.6, *P* < 0.001) (Table 2). In patients with complications, they had higher incidence of preoperative comorbidities such as coronary heart disease (*P* = 0.023), pulmonary disease (*P* = 0.024), and mild cognitive dysfunction (*P* < 0.001) (Table 2). Preoperative albumin was lower in patients with complications than in patients without complications (40.0 ± 4.6, vs. 41.0 ± 4.7, *P* = 0.001) (Table 2).

**Table 2:** Baseline characteristics.

**Variable**	**All patients (*n* = 915)**	**Non-complication group (*n* = 496)**	**Complication group (*n* = 419)**	***P***
Age, year, mean ± SD	71.6 ± 5.2	70.7 ± 4.6	72.7 ± 5.7	<0.001
≥75 years old, *n* (%)	264 (28.9%)	109 (22.0%)	155 (37%)	<0.001
Male, *n* (%)	548 (59.9%)	309 (62.3%)	239 (57.0%)	0.106
BMI, kg/m^2^, mean ± SD	24.2 ± 3.5	24.2 ± 3.4	24.1 ± 3.6	0.783
**Preoperative comorbidity**, ***n*** **(%)**				
Stroke	52 (5.7%)	28 (5.6%)	24 (5.7%)	0.957
Hypertension	475 (51.9%)	252 (50.8%)	223 (53.2%)	0.466
Coronary heart disease	129 (14.1%)	58 (11.6%)	71 (16.9%)	0.023
Arrhythmia	57 (6.2%)	24 (4.8%)	33 (7.9%)	0.058
Pulmonary disease^**a**^	66 (7.2%)	27 (5.4%)	39 (9.3%)	0.024
Diabetes	219 (23.9%)	112 (22.6%)	107 (25.5%)	0.296
Hyperlipidemia	95 (10.4%)	48 (9.6%)	47 (11.2%)	0.447
Hepatic dysfunction, *n* (%)^**b**^	45 (4.9%)	19 (3.8%)	26 (6.2%)	0.098
Malignant tumor, *n* (%)^**c**^	105 (11.5%)	53 (10.7%)	52 (12.4%)	0.415
Chronic smoking, *n* (%)^**d**^	223 (24.4%)	128 (25.8%)	95 (22.7%)	0.271
CCI, median (IQR)	2 (2, 3)	2 (2, 3)	2 (2, 3)	0.102
Mild cognitive dysfunction, *n* (%)^**e**^	597 (65.2%)	286 (57.7%)	311 (74.2%)	<0.001
PONS, median (IQR)	0 (0, 1)	0 (0, 0)	0 (0, 1)	<0.001
PONS ≥ 1, *n* (%)	250 (27.3%)	90 (18.1%)	160 (38.2%)	<0.001
NRS2002, median (IQR)	3 (2, 4)	2 (2, 3)	3 (2, 4)	<0.001
NRS2002 ≥ 3, *n* (%)	490 (53.6%)	235 (47.4%)	255 (60.9%)	<0.001
**ASA classification**, ***n*** **(%)**				
II	678 (74.0%)	395 (79.6%)	283 (67.5%)	
III	237 (26.0%)	101 (20.4%)	136 (32.5%)	
**Laboratory tests**				
Albumin (g/L)	40.6 ± 4.7	41.0 ± 4.7	40.0 ± 4.6	0.001
<30 g/L, *n* (%)	22 (2.4%)	12 (2.4%)	10 (2.4%)	0.974
Creatinine (μmol/L)	80.2 ± 20.9	79.5 ± 19.9	81.0 ± 22.0	0.300
Glucose, mmol/L, mean ± SD	6.0 ± 1.7	6.0 ± 1.6	6.0 ± 1.9	0.832

*SD, standard deviation; BMI, body mass index; CCI, Charlson Comorbidity Index; PONS, perioperative nutrition screen; IQR, interquartile range; NRS2002, Nutritional Risk Screening 2002; ASA, American Society of Anesthesiology*.

a *Pulmonary disease included chronic obstructive pulmonary disease and asthma*.

b *Hepatic dysfunction was defined as alanine transaminase and/or aspartate transaminase higher than five times the upper normal limit*.

c *Malignant tumor was defined as carcinoma (carcinoma, squamous cell carcinoma, and adenocarcinoma), sarcoma, and undifferentiated carcinoma*.

d *Chronic smoking was defined as half a pack of cigarettes per day for at least 2 years*.

e *Mild cognitive dysfunction was defined as Montreal cognitive assessment score <27*.

Patients with complications experienced higher volume of blood loss (*P* < 0.001), received more allogeneic blood transfusion (*P* < 0.001), and had prolonged surgery time (*P* < 0.001) (Table 3).

**Table 3:** Perioperative variables.

**Variable**	**All patients (*n* = 915)**	**Non-complication group (*n* = 496)**	**Complication group (*n* = 419)**	***P***
Type of anesthesia, *n* (%)				0.262
General anesthesia	420 (45.9%)	229 (46.2%)	191 (45.6%)	
General-PNB anesthesia^**a**^	469 (51.3%)	257 (51.8%)	212 (50.6%)	
Epidural-general	26 (2.8%)	10 (2.0%)	16 (3.8%)	
**Intraoperative medication**				
Use of nitrous oxide, *n* (%)	553 (60.4%)	299 (60.3%)	254 (60.6%)	0.917
Use of sevoflurane, *n* (%)	287 (31.4%)	150 (30.2%)	137 (32.7%)	0.425
Use of dexmedetomidine, *n* (%)	430 (47.0%)	232 (46.8%)	198 (47.3%)	0.884
Use of midazolam, *n* (%)	189 (20.7%)	105 (21.2%)	84 (20.0%)	0.676
Use of etomidate, *n* (%)	699 (76.4%)	367 (74.0%)	332 (79.2%)	0.063
Propofol (mg) median (IQR)	840 (642, 1,075)	800 (620, 1,040)	875 (680, 1,132)	<0.001
Sufentanil equivalent (μg)^b^ median (IQR)	110 (77, 160)	109 (75, 158)	80 (112, 165)	0.162
Surgery time (h), mead ± SD	3.4 ± 1.2	3.3 ± 1.1	3.5 ± 1.2	0.001
Location of surgery, *n* (%)				0.296
Intra-thoracic	198 (21.6%)	114 (23.0%)	84 (20.0%)	
Intra-abdominal	530 (57.9%)	289 (58.3%)	241 (57.5%)	
Spinal/extremities/others	187 (20.4%)	93 (18.8%)	94 (22.4%)	
Estimated blood loss, ml, median (IQR)	100 (10, 250)	50 (10, 200)	100 (10, 300)	<0.001
Allogeneic blood transfusion, *n* (%)	79 (8.6%)	27 (5.4%)	52 (12.4%)	<0.001
Total fluid infusion (ml), median (IQR)	2,200 (1,600, 2,850)	2,100 (1,600, 2,600)	2,350 (1,750, 3,100)	<0.001
Urine output (ml), median (IQR)	400 (250, 600)	250 (400, 600)	350 (200, 600)	0.632
Use of parenteral nutrition, *n* (%)	343 (37.5%)	168 (33.9%)	175 (41.8%)	0.016
Postoperative LOS, days, median (IQR)	8 (6, 11)	7 (5, 9)	9 (6, 12)	<0.001

*PNB, peripheral nerve block; SD, standard deviation; IQR, interquartile range; LOS, length of in-hospital stay*.

a *Combined general-PNB anesthesia indicated that patients received both general anesthesia and peripheral nerve block (including epidural anesthesia)*.

b *Intraoperative opioids included remifentanil and sufentanil. The dosage of remifentanil was converted into equivalent of sufentanil according to the following formula: dosage of sufentanil (μg) = the dosage of remifentanil (μg)/10*.

### Perioperative Confounding Factors of Postoperative Complications

Univariate analysis was firstly used to screen potential risk factors of postoperative complications from baseline and perioperative variables. Variables with P < 0.05 were then entered into multivariable analysis (i.e., age, coronary heart disease, pulmonary disease, ASA classification, mild cognitive dysfunction, duration of surgery, and allogeneic blood transfusion) (Table 4).

**Table 4:** The relationship between PONS and postoperative complications.

**Variables**	**Univariate analysis**			**Multivariate analysis PONS score** **≥** **1**^**a**^			**Multivariate analysis NRS2002 score** **≥** **3**^**a**^		
	**OR**	**95% CI**	**P**	**OR**	**95% CI**	**P**	**OR**	**95% CI**	**P**
Age (per year increase)	1.076	1.048–1.104	<0.001	1.059	1.029–1.089	<0.001	1.055	1.024–1.087	<0.001
Coronary heart disease (yes)	1.541	1.060–2.240	0.024	—	—	—	1.149	0.754–1.752	0.519
Pulmonary disease (yes)^**b**^	2.043	1.220–3.422	0.007	2.088	1.205–3.619	0.009	—	—	—
Mild cognitive dysfunction^**c**^	2.114	1.595–2.804	<0.001	2.100	1.557–2.833	<0.001	2.083	1.553–2.794	<0.001
ASA classification (per grade increase)	1.879	1.393–2.535	<0.001	1.399	1.009–1.939	0.044	1.523	1.108–2.092	0.009
NRS2002 score ≥ 3	1.727	1.327–2.248	<0.001	NA	NA	NA	1.313	0.973–1.771	0.075
PONS score ≥ 1	2.787	2.061–3.768	<0.001	2.308	1.676–3.178	<0.001	NA	NA	NA
Duration of surgery (per hour increase)	1.203	1.074–1.348	0.001	1.149	1.014–1.302	0.029	1.212	1.073–1.369	0.002
Allogeneic blood transfusion (yes)	2.461	1.516–3.996	<0.001	2.119	1.259–3.567	0.005	2.091	1.250–3.497	0.005

*ASA, American Society of Anesthesiologists; NRS2002, Nutritional Risk Screening 2002; PONS, perioperative nutrition screen; OR, odds ratio; CI, confidence interval; NA, not available*.

a *PONS ≥ 1 and NRS2002 ≥ 3 were put into the multivariate analysis separately*.

b *Pulmonary disease was defined as chronic obstructive pulmonary disease and asthma*.

c *Mild cognitive impairment was defined as Mini-Mental State Examination (MMSE) score <27*.

### The Relationship Between Perioperative Nutrition Screen and Postoperative Complications

According to PONS ≥ 1, 27.3% (250/915) patients were at risk of malnutrition. PONS ≥ 1 was associated with an increased risk of postoperative complications in both univariate analysis (OR 2.787, 95% CI 2.061–3.768, *P* < 0.001) and multivariable analysis (OR 2.308, 95% CI 1.676–3.178, *P* < 0.001) after adjusting for the above confounding factors.

### The Relationship Between Nutritional Risk Screening 2002 and Postoperative Complications

According to NRS2002 ≥ 3, 53.6% (490/915) patients were at risk of malnutrition. NRS2002 ≥ 3 was associated with an increased risk of postoperative complications in univariate analysis (OR 1.727, 95% CI 1.327–2.248, *P* < 0.001), but not in multivariable analysis (OR 1.313, 95% CI 0.973–1.771, *P* = 0.075) after adjusting the above confounders (Table 4).

### Predictive Performance of Perioperative Nutrition Screen and Nutritional Risk Screening 2002 Against Postoperative Complications

**Figure 2:**
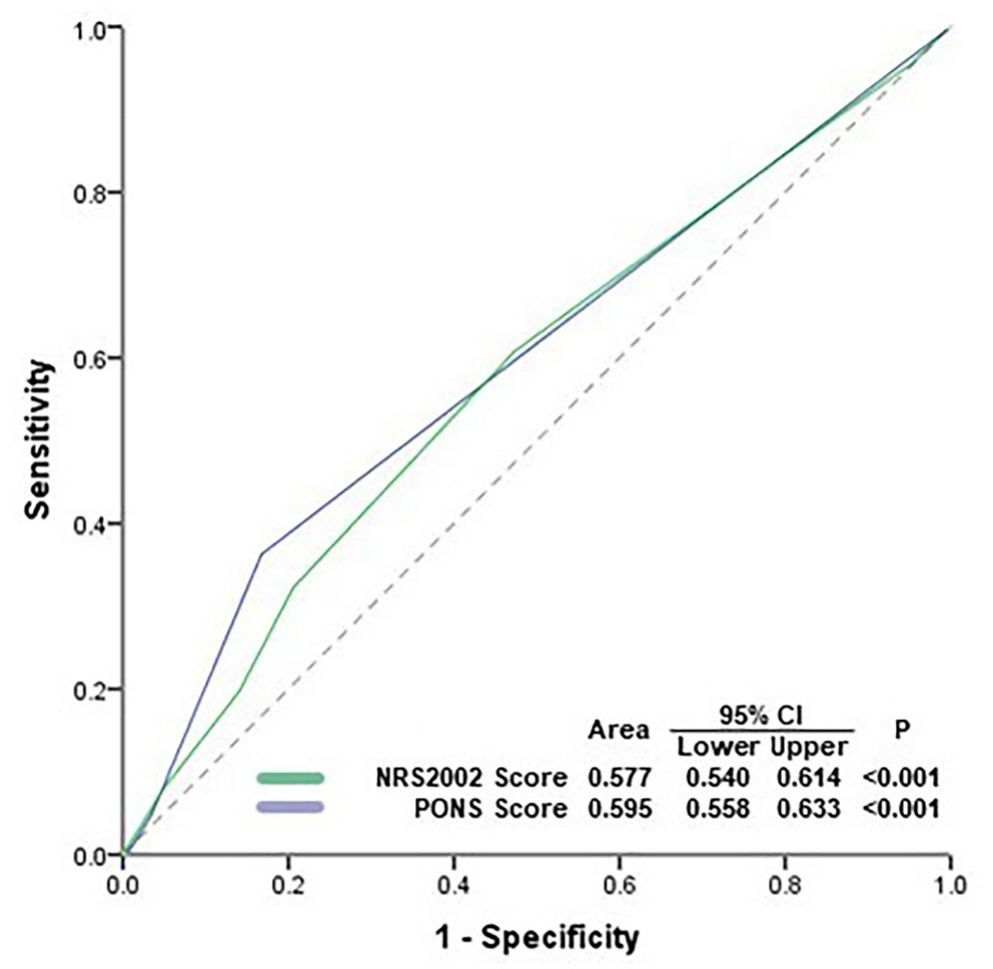
Receiver operating characteristic (ROC) analysis. ROC curve analysis showed that the performances of PONS and NRS2002 were poor in predicting postoperative complications. PONS, perioperative nutrition screen; NRS2002, Nutritional Risk Screening 2002; AUC, area under curve; CI, confidence interval.

ROC curve analysis showed that the performances of PONS [area under the ROC curve (AUC) 0.595, 95% CI 0.558–0.633] and NRS2002 (AUC 0.577, 95% CI 0.540–0.614) were poor in predicting overall postoperative complications (Figure 2).

## Discussion

The present study found that malnutrition diagnosed by PONS was related with an increased risk of postoperative complications. However, use of PONS in predicting postoperative complications requires more attention because its performance may be affected by the type of complications.

Perioperative malnutrition has raised up more and more attention because it is highly related with poor patient outcome (1, 2, 10). Its clinical manifestation varies greatly in surgical patients and mainly includes lower BMI, body weight loss, hypoalbuminemia, decrement of oral intake, and microelement insufficiency (1, 2, 10). There are emerging studies to support that preoperative lower BMI is an independent predictor of postoperative complications such as in patients with gastric cancer resection and hip surgery (20, 21). A prospective study including 331 cardiac surgery patients shows that preoperative unintended weight loss more than 10% baseline is related with increased risk of postoperative infections and prolonged stay in the intensive care unit (22). In patients undergoing spinal surgery, hypoalbuminemia is associated with increased risk (about two to five times) of postoperative 30-day mortality and complications with a dose-dependent effect (23). Microelement (i.e., magnesium and vitamin D) insufficiency is common in surgical patients and highly related with increased mortality and complications (such as delirium, postoperative cognitive dysfunction, and infection) (24, 25).

Beyond the miscellaneous symptoms listed above, definition and diagnosis of malnutrition are still a major concern in clinical practice (1, 2, 10). The inconsistency in malnutrition criteria may lead to vast differences in its incidence and incomparability between clinical trials. For example, the incidence of malnutrition was 27.3% by PONS and 53.6% by NRS2002 in the present study. Compared with PONS, NRS2002 includes more items for diagnosis criteria, which may increase the incidence of malnutrition (1, 15). First, NRS2002 takes severity of disease as an important predictor if the patient will experience impaired food intake and increased stress metabolism during the forthcoming clinical treatment. Second, the criteria of abnormal body weight loss and oral intake are easier to achieve in NRS2002 than in PONS. For example, oral intake of 50–75% normal requirement is considered as mild deficit in NRS2002, whereas oral intake <50% baseline was considered as abnormal in PONS. Third, NRS2002 considers age ≥70 years as additional score.

Although PONS has been recommended by American Society for Enhanced Recovery and Perioperative Quality Initiative Joint Consensus Statement, there are no sufficient data to elucidate its relationship with postoperative complications (1). In a validation study of 273 patients, the incidence of malnutrition based on PONS criteria among surgical patients was about 27%, which was consistent with the present study (18). But it did not examine the relationship between malnutrition and postoperative complications. The present study found that PONS was associated with increased risk of complications within postoperative 30 days in elderly surgical patients.

In the present study, we found that both PONS score and NRS2002 score performed poorly in predicting postoperative complications by ROC analysis. First, it seems that malnutrition may have different impact on the occurrence of complications in different systems. In the present study, the AUC of PONS score in predicting surgery-related complications and infection was 0.803 (95% CI 0.715–0.891) and 0.754 (95% CI 0.656–0.852), respectively, but the AUC in predicting cardiovascular complications was only 0.611 (95% CI 0.517–0.704). However, we did not present the *post-hoc* analysis in the present report because the individual incidences of these complications were merely about 1–5% and the sample size could not provide sufficient statistical power.

Multivariable analysis showed that age, pulmonary disease, mild cognitive dysfunction, ASA classification, duration of surgery, and allogeneic blood transfusion were related with postoperative complications. These findings were in accordance with previous studie (26–31). It indicated that the underlying disease of patients and intrinsic risk of surgical procedures also played important roles in the development of postoperative complications.

This study has two limitations. First, our result was generated from a single-center study, which might limit its generality. Second, the 30-day mortality was about 1.4% in the present study, and the sample size was insufficient to analyze the relationship between malnutrition and mortality.

## Conclusion

The present study found that malnutrition diagnosed by PONS was related with an increased risk of postoperative complications. However, use of PONS in predicting postoperative complications requires more attention because its performance may be affected by the type of complications.

## Data Availability Statement

The raw data supporting the conclusions of this article will be made available by the authors, without undue reservation.

## Ethics Statement

Ethical approval for this study was provided by the Clinical Research Ethics Committee of Peking University First Hospital (Chairperson Prof Guo Xiaohui) on August 4, 2017 [2017 (1419), Beijing, China], and registered with Chinese Clinical Trial Registry on September 19, 2017 (ChiCTR-OOC-17012734). Written informed consent was obtained from all participants or their legal representatives.

## Author Contributions

FZ helped in data acquisition, data analysis, and manuscript drafting. S-TH helped in data acquisition and data analysis. YZ helped in data acquisition and data analysis. D-LM helped in concept and design, data analysis, data interpretation, manuscript drafting, and final approval of submission. D-XW helped in concept and design, administrative or material support, and critical revision of the manuscript for important intellectual content. All authors contributed to the article and approved the submitted version.

## Conflict of Interest

The authors declare that the research was conducted in the absence of any commercial or financial relationships that could be construed as a potential conflict of interest.
